# Variability in the Risk of Malignancy in Bethesda IV Thyroid Nodules: Results from the EUROCRINE Surgical Registry

**DOI:** 10.3390/cancers18193186

**Published:** 2026-10-02

**Authors:** Piotr Wiśniewski, Maciej Śledziński, Marcin Barczyński, Andrzej Rafał Hellmann

**Affiliations:** 1 Department of Endocrinology and Internal Diseases, Medical University of Gdańsk, 80-214 Gdansk, Poland; piotr.wisniewski@gumed.edu.pl; 2 Department of Surgical Oncology, Transplant Surgery and General Surgery, Medical University of Gdańsk, 80-214 Gdansk, Poland; maciej.sledzinski@gumed.edu.pl; 3 Faculty of Medicine, Department of Endocrine Surgery, Jagiellonian University Medical College, 31-501 Krakow, Poland; marbar@mp.pl

**Keywords:** Bethesda IV, risk of malignancy, EUROCRINE registry, thyroid nodules, follicular neoplasm

## Abstract

**Background/Objectives:** The management of Bethesda IV (follicular neoplasm) thyroid nodules remains controversial, in part due to wide variation in the reported risk of malignancy (ROM-BIV). Understanding this variability is essential for documenting systemic inconsistencies in ROM-BIV estimates and for developing more consistent risk-stratification strategies. This study aimed to quantify ROM-BIV variability across European centers and conduct an exploratory analysis of the trade-offs associated with hypothetical risk-based thresholds within a surgically selected cohort. **Methods:** We analyzed data from 15,251 patients with Bethesda IV thyroid nodules enrolled in the EUROCRINE registry. Multilevel logistic regression models were used to estimate ROM-BIV and assess variation across centers and countries, adjusting for patient- and nodule-level characteristics. Threshold analysis and decision curve analysis were employed as illustrative mappings of trade-off mechanisms within the surgically treated cohort to explore how varying risk levels affect classification outcomes. **Results:** Adjusted center-level ROM-BIV demonstrated marked variability, ranging from 5.5% to 65.5%. Intraclass correlation coefficients (ICCs) were 11.4% for institutions and 9.7% for countries, indicating meaningful clustering of ROM-BIV at both levels. The fixed component of the model showed limited-to-moderate discrimination (AUC-ROC 0.647; 95% CI, 0.637–0.656). Exploratory analysis illustrated the inherent trade-off between reducing potential overdiagnosis and maintaining sensitivity for malignancy detection. Decision curve analysis indicated a theoretical net benefit over default strategies, although no specific clinical threshold could be recommended based on these retrospective data. **Conclusions:** Substantial variability in ROM-BIV across European centers may reflect differences in institutional selection, diagnostic practices, clinical management, and reporting, rather than biological variance. These findings highlight the need for standardization of diagnostic and reporting pathways. Risk-based models must be positioned as supportive tools for individualized management and require rigorous prospective validation in unselected populations before any clinical implementation can be considered.

## Simple Summary

Bethesda IV thyroid nodules remain a clinical challenge because cytology alone cannot reliably distinguish benign from malignant follicular-patterned lesions, and many patients therefore undergo diagnostic surgery for nodules that ultimately prove benign. Using data from the EUROCRINE surgical registry, we analyzed 15,251 operated patients with Bethesda IV nodules and found that the risk of malignancy varied markedly across European centers and countries, even after adjustment for available patient- and ultrasound-related factors. Age and EU-TIRADS category were associated with malignancy risk, whereas institutional surgical volume was not. Our findings suggest that local diagnostic interpretation and treatment selection may contribute to the observed variation in the risk of malignancy. Exploratory model-based analyses suggest that risk-adapted strategies may help inform future decision-making, but they require prospective validation before clinical implementation.

## 1. Introduction

Thyroid nodules are diagnosed with increasing frequency and are among the most common endocrine findings in adult populations [1,2]. Their evaluation begins with clinical and ultrasound risk stratification, which guides the decision to perform fine-needle aspiration (FNA). The resulting cytological classification, using the Bethesda System for Reporting Thyroid Cytopathology, estimates the risk of malignancy and guides further management [3,4]. In typical cytology series, most nodules are categorized as benign, often approximately 60–70% (Bethesda class II), whereas malignant (Bethesda class VI) and indeterminate categories (Bethesda classes III, IV, and V) account for smaller proportions; however, these estimates vary depending on the population studied, referral patterns, FNA indications, and local cytopathology practice [5].

Bethesda category IV, denoting a “follicular neoplasm”, represents a diagnostic gray zone where current non-invasive diagnostics cannot reliably distinguish between benign and malignant follicular lesions. As a result, Bethesda IV nodules have traditionally been managed with diagnostic surgery, usually lobectomy, to obtain a definitive tissue diagnosis, although many ultimately prove benign. This creates an important clinical dilemma, as patients with benign final histopathology are exposed to the burden of surgery without oncological benefit, including permanent scarring, potential complications, and possible difficulty during future neck reoperation. These limitations underscore the need for improved, preferably less invasive, preoperative risk stratification.

Clinical guidelines generally consider diagnostic surgery an important management option [6,7]. Molecular testing may be considered where available, and surveillance may be an option in carefully selected small, low-risk nodules [6]. While this introduces some degree of individualized care, a fully risk-adapted approach based on quantitative malignancy estimates is not yet standard, and management may vary across institutions.

Although the average risk of malignancy in surgically treated Bethesda IV thyroid nodules (ROM-BIV) has been estimated at approximately 30%, published literature demonstrates significant variation in ROM-BIV rates between different centers and countries, ranging from 15% to 50% [4,8,9,10]. This variability may reflect differences in study design, patient selection, and diagnostic practices (e.g., diagnostic criteria, surgical thresholds, study period, and classification of borderline entities such as NIFTP). This considerable variability contributes to clinical uncertainty and may substantially influence management decisions. For instance, active surveillance might be considered acceptable in selected patients when the expected malignancy risk is close to 15%, whereas a risk approaching 50% would more strongly favor surgical intervention.

The aim of this study was twofold: (1) to quantify the institutional and between-country heterogeneity in ROM-BIV within a surgically selected cohort from the EUROCRINE registry, and (2) to conduct an exploratory analysis of the trade-off mechanisms between sensitivity and surgical selection using hypothetical risk thresholds. By documenting the extent of systemic variability, this study seeks to provide a foundational argument for the standardization of diagnostic and reporting pathways, rather than offering a validated tool for clinical decision-making. This assessment is intended to support, rather than replace, individualized management by highlighting the context-dependent nature of malignancy risk in Bethesda IV nodules.

## 2. Materials and Methods

### 2.1. Data Source

The present study was approved by the Independent Bioethics Committee for Scientific Research of the Medical University of Gdansk (NKBBN/407/2025, approval date 12 September 2025). A retrospective analysis was performed using data from the European Registry for Thyroid Surgery (EUROCRINE). EUROCRINE is a voluntary surgical registry, and the completeness of thyroid surgery reporting at the center, country, or European level could not be verified. The registry captures essential information including patient demographics, preoperative diagnostic findings (ultrasound malignancy risk, EU-TIRADS class [3], cytological Bethesda class [4]), surgical details, final histopathological diagnosis, and postoperative complications [11]. For the present analysis, thyroid surgeries performed between 2015 and 2023 were considered.

### 2.2. Outcome and Covariates

Thyroid surgeries were included if the preoperative cytological diagnosis was Bethesda category IV and the final histopathological diagnosis was available. Institutions that submitted fewer than 5 eligible Bethesda IV cases were excluded from the analysis (20 centers; 44 cases). This pragmatic threshold was chosen after inspection of reporting patterns in EUROCRINE, as centers below this threshold generally represented sporadic or discontinued registry participation. The exclusion was intended to reduce instability related to transient reporting, rather than to exclude low-volume centers on clinical grounds.

The outcome of interest was the presence of malignant thyroid neoplasm as reported in the final pathology diagnosis (recorded as the main histological diagnosis). Non-invasive follicular thyroid neoplasm with papillary-like nuclear features (NIFTP) and tumors of uncertain malignant potential (UMP) were considered borderline tumors warranting surgical removal and follow-up and were excluded from both the malignant and benign categories.

The covariates considered in the main model were registry-available variables selected a priori: sex, age, EU-TIRADS category, and center volume. Center volume was defined pragmatically based on the number of thyroid operations reported to EUROCRINE per year. Centers reporting ≥75 thyroid operations annually were classified as higher-volume centers, whereas those below this threshold were classified as lower-volume centers. This analytical cut-off was used to distinguish centers with greater versus lower registry-reported experience and should not be interpreted as a universal definition of a high-volume thyroid surgery center. Exclusion of centers with fewer than five eligible cases reduced instability from sporadic reporting but may have influenced representativeness.

Several potentially relevant variables, including nodule number, molecular testing, comorbidities, family history of thyroid cancer, prior radiation exposure, and surgeon specialty, were unavailable or not captured in a sufficiently consistent or structured manner for inclusion. Nodule size was available only for a minority of patients and was therefore considered too incomplete for reliable inclusion as a predictor of malignancy.

### 2.3. Statistical Methods

Missing data were handled using multiple imputation by chained equations. EU-TIRADS category was the only variable included in the main multivariable model with missing data (7701/15,251; 50.5%) and was imputed using multinomial logistic regression. Five imputed datasets were generated with 10 iterations. The imputation model included malignancy status, sex, age spline terms, and center volume category; institution and country identifiers were retained for the multilevel model but were not used as predictors in the imputation model. Model estimates were pooled according to Rubin’s rules. The proportion of missing data for variables included in the main model is reported in Supplementary Table S3. Descriptive and inferential statistics were based on the imputed datasets.

A three-level hierarchical logistic regression model was fitted for malignancy on final histopathological diagnosis, as defined above. Fixed effects included sex, a restricted cubic spline for age (3 degrees of freedom), EU-TIRADS category, and center volume. Random intercepts were specified for surgical centers, nested within countries. The model was estimated using maximum likelihood with Laplace approximation. Predicted probabilities with random effects were used to estimate adjusted center- and country-level ROM-BIV, whereas marginal predictions without random effects were used for threshold-based analyses and decision curve analysis. Because the model was derived from a surgically selected cohort, these predictions should not be interpreted as generalizable patient-level risk estimates for all Bethesda IV nodules. Residual center- and country-level heterogeneity may partly reflect unmeasured factors such as nodule size, multifocality, molecular testing, and temporal changes in practice.

To illustrate the consequences of applying hypothetical risk thresholds, we conducted an exploratory decision curve analysis (DCA). DCA compares the net benefit of alternative classification strategies while accounting for the relative consequences of false-positive and false-negative classifications [12]. In this analysis, the target population comprised surgically treated Bethesda IV thyroid nodules, and the intervention was diagnostic surgery for definitive histopathological diagnosis. The model-based strategy was compared with two default strategies: classifying all patients or no patients as above threshold. The decision threshold was defined as the minimum predicted ROM-BIV required for classification as above threshold. Net benefit was evaluated over thresholds of 15–50%, selected to illustrate the behavior of the model across a broad range of hypothetical risk levels.

Model performance was assessed internally using AUC-ROC, calibration across predicted-risk deciles, calibration intercept and slope, and Brier score. No external validation or cross-validation was performed; threshold-based and decision-curve analyses were therefore considered exploratory. All statistical analyses were conducted using R version 4.5.3 (R Foundation for Statistical Computing, Vienna, Austria) and the mice 3.19.0, lme4 1.1-38 and dcurves 0.5.1 packages [13,14,15,16].

## 3. Results

### 3.1. Study Population and Baseline Characteristics

A total of 114 surgical centers from 10 countries contributed data to the analysis, comprising 15,251 operations. The number of included cases per country ranged from 36 to 8340, and per center from 5 to 7024. The median number of cases per center was 36 (IQR 14–70). Characteristics of the participants are presented in Table 1.

### 3.2. Overall ROM-BIV and Model Predictors

The overall ROM-BIV within this surgically selected cohort was 30%. Because EUROCRINE captures surgically treated patients, this estimate represents ROM-BIV among selected operated nodules, not population-level ROM-BIV. Substantial variation was observed between institutions (IQR 21–39%) and countries (IQR 24–34%).

To explore whether differences in case mix contributed to institutional ROM-BIV heterogeneity, we compared patients treated in centers from the lowest and highest tertiles of crude institutional ROM-BIV (Supplementary Table S4). High-ROM centers had a higher proportion of total thyroidectomies than low-ROM centers (44.9% vs. 21.3%) and, by definition, a higher proportion of malignant final histology (47.8% vs. 16.2%). The distribution of EU-TIRADS categories was broadly similar between groups, whereas malignant tumors were smaller in high-ROM centers (median 14 mm vs. 24 mm). These findings are consistent with differences in surgical case mix, selection thresholds, and pathology spectrum across centers, although their individual contributions could not be quantified without data on non-operated Bethesda IV nodules.

**Figure 1:**
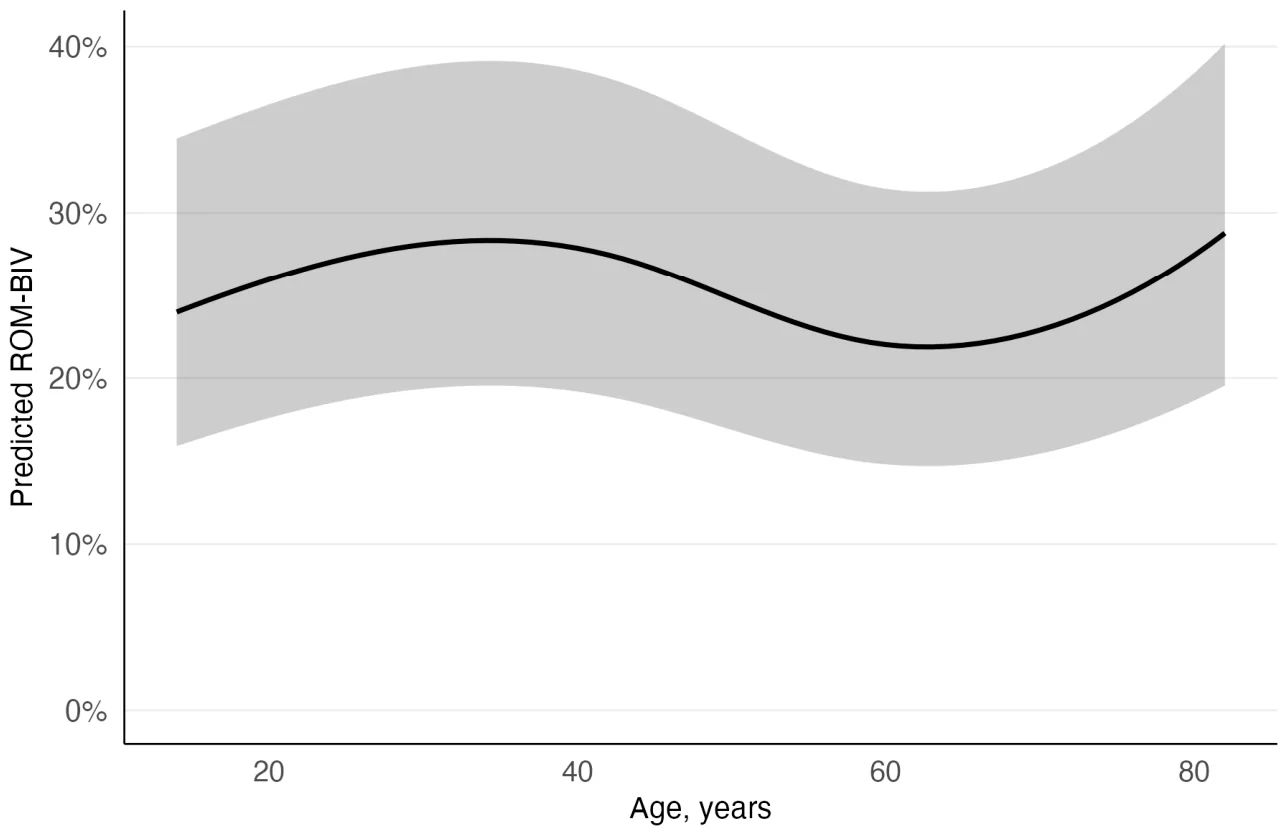
Predicted ROM-BIV according to age, based on the fixed-effects component of the multilevel logistic model, pooled across imputed datasets. Shaded area represents the 95% confidence interval. Age range restricted to the 1st–99th percentile.

In the final multilevel logistic regression model (Table 2), age demonstrated a statistically significant non-linear association with ROM-BIV, with all spline terms reaching significance. The resulting U-shaped relationship indicated higher malignancy risk among younger and older patients, and lower risk in middle-aged individuals (Figure 1). Higher EU-TIRADS categories were strongly associated with increased malignancy risk, with stepwise increases observed from TR4 to TR5 compared to TR1–3. The observed U-shaped relationship between age and ROM-BIV is strictly descriptive for this operated cohort and likely reflects age-dependent clinical triage and surgical selection thresholds. We did not observe an independent association between female sex or the pragmatic center-volume category and malignancy after adjustment.

### 3.3. Institutional and Country-Level Heterogeneity

The model included random intercepts for institutions nested within countries, capturing hierarchical clustering of ROM-BIV. Averaged across the imputed datasets, the variance of the random intercepts was estimated at 0.48 for centers and 0.41 for countries, corresponding to standard deviations of 0.69 and 0.64, respectively. On the latent logistic scale, these values corresponded to ICCs of 11.4% for centers and 9.7% for countries, with stable estimates across imputations: 11.3–11.5% for centers and 9.6–9.8% for countries. Together, these findings indicate that a meaningful proportion of the variability in observed ROM-BIV was attributable to clustering at the institutional and country levels.

**Figure 2:**
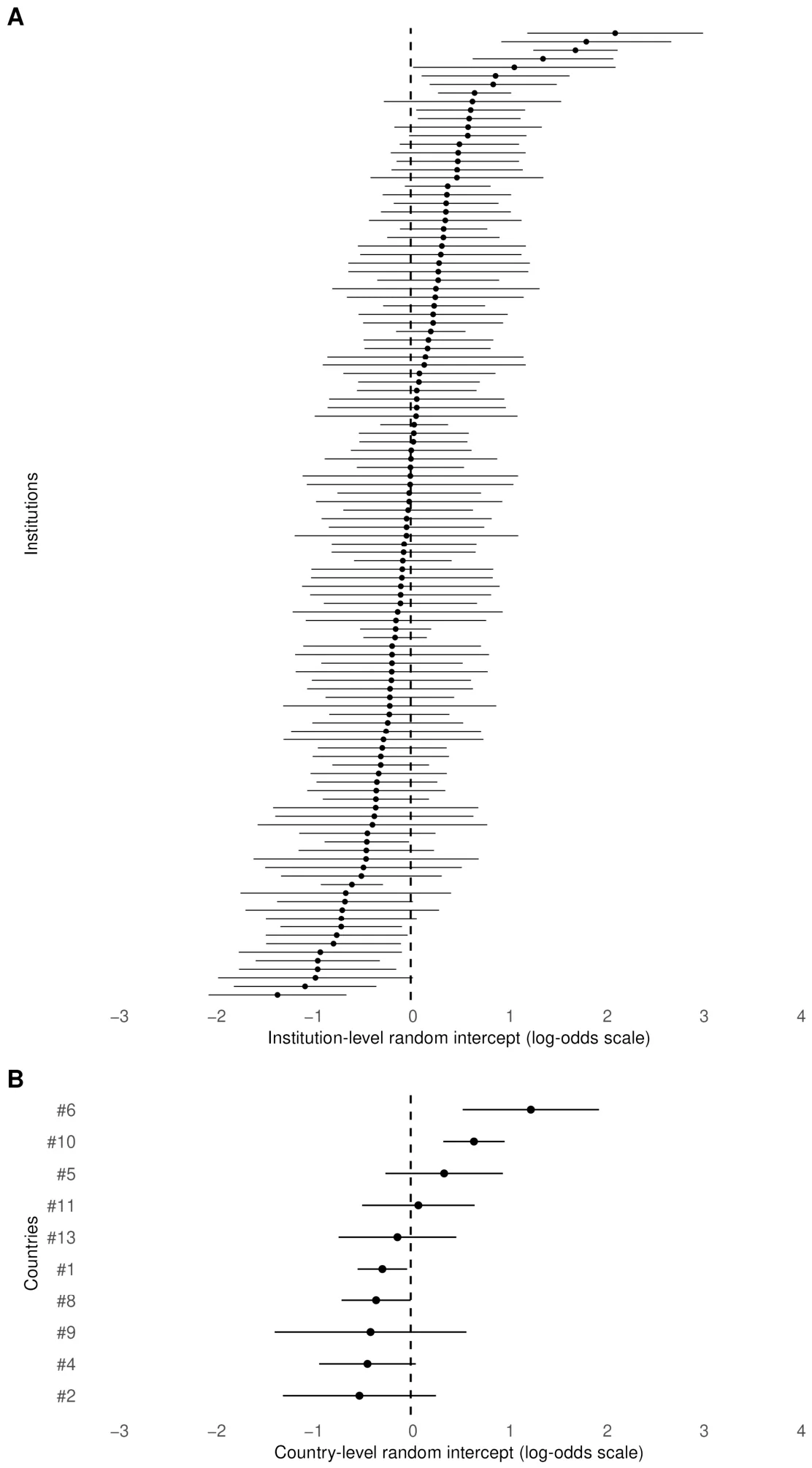
Institution- and country-level random intercepts for ROM-BIV. Panel (**A**) shows institutions (*n* = 114) and panel (**B**) shows countries (*n* = 10), ordered by estimated random intercept. Points represent estimates on the log-odds scale; horizontal lines represent 95% confidence intervals pooled across imputed datasets. Positive values indicate higher adjusted ROM-BIV than the overall average.

Institution-level random intercepts varied widely across centers, ranging from −1.37 to +2.11 on the log-odds scale (Figure 2A), indicating substantial residual institutional heterogeneity after adjustment for measured covariates. Corresponding adjusted center-level ROM-BIV estimates ranged from 5.5% to 65.5% (Supplementary Table S5). Because extreme center-level estimates may be influenced by small sample size and local selection practices, we performed sensitivity analyses excluding small centers and centers with extreme crude ROM-BIV values (Supplementary Table S5). Excluding centers with fewer than 20 or 50 patients had little effect on crude ROM-BIV and did not eliminate between-center variability. Excluding centers below the 5th or above the 95th percentile of crude ROM-BIV narrowed the adjusted center-level ROM-BIV range, although variability persisted. These findings suggest that the most extreme estimates were partly driven by outlying centers, while substantial institutional heterogeneity remained.

Between-country variability was more limited than between-center variability. Country-level log-odds estimates ranged from approximately −0.53 to +1.24 on the log-odds scale, indicating additional between-country heterogeneity after adjustment for measured covariates. Four countries had 95% confidence intervals that did not cross zero, indicating statistically significant deviation from the overall mean on the log-odds scale (Figure 2B). Because one anonymized country contributed disproportionately to the cohort, we additionally repeated the sensitivity analysis after excluding Country 10. This reduced the overall crude ROM-BIV from 30.7% to 26.9%, but substantial adjusted center-level variability persisted, with center-level ROM-BIV ranging from 10.6% to 74.2% (Supplementary Table S5).

### 3.4. Internal Model Performance

The model explained only a modest proportion of the overall variance. The marginal R^2^ was 0.009, while the conditional R^2^ was 0.219, with stable estimates across imputations (marginal R^2^ range 0.008–0.011; conditional R^2^ range 0.217–0.220), indicating that fixed effects explained little ROM-BIV variability, whereas institution- and country-level random effects increased the explained variance. This suggests that a substantial proportion of the observed variability was not explained by the measured patient- and nodule-level characteristics and may reflect unmeasured institutional, diagnostic, and selection-related factors.

The fixed-effects-only marginal prediction model showed limited-to-moderate discrimination, with an AUC-ROC of 0.647 (95% CI 0.637–0.656). Calibration was good, with a calibration intercept of 0.000, calibration slope of 1.14, and Brier score of 0.196; predicted and observed malignancy rates aligned closely across deciles (Supplementary Figure S1).

**Figure 3:**
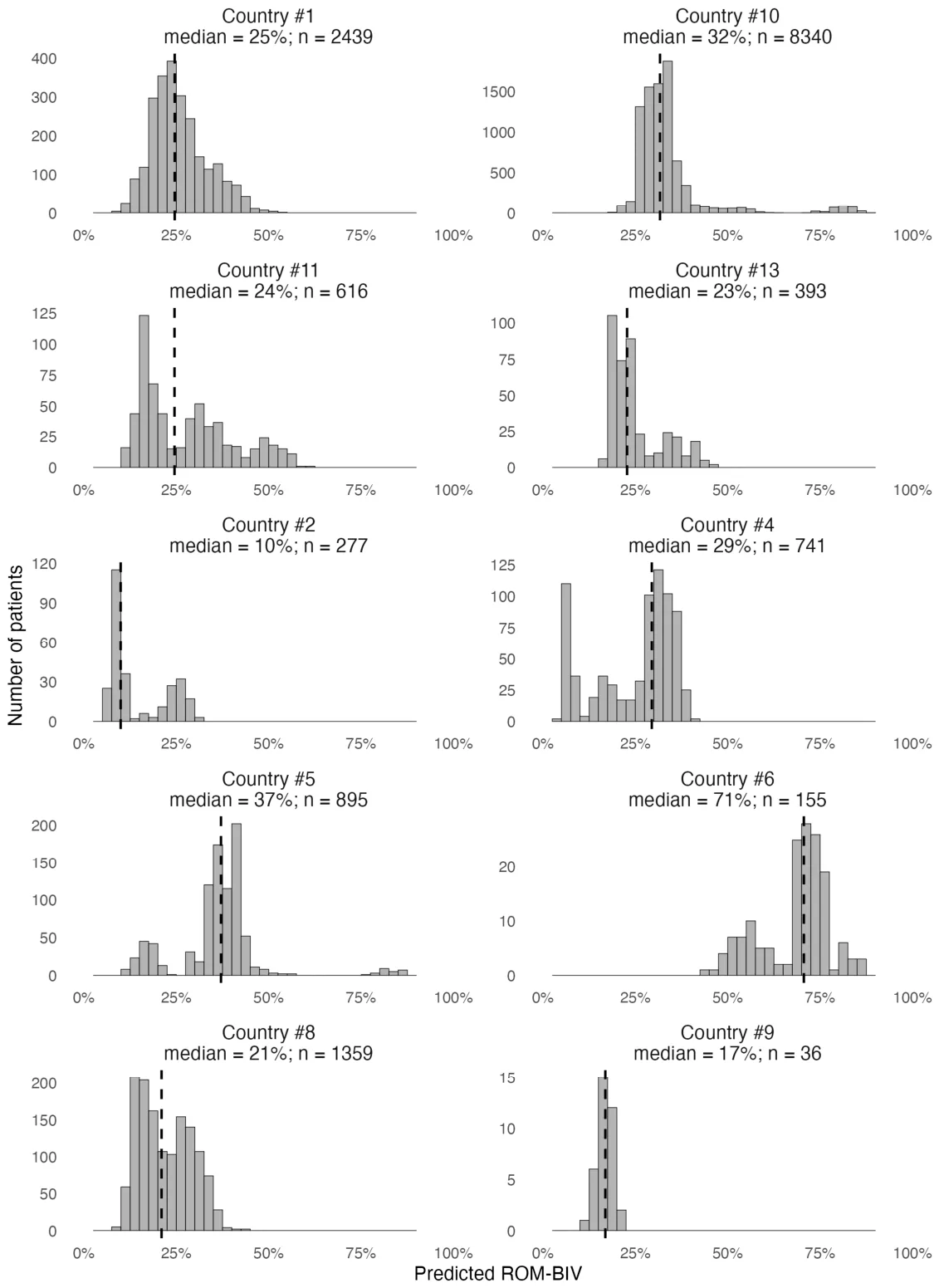
Distribution of predicted ROM-BIV by country. Histograms show patient-level predicted probabilities averaged across imputed datasets. Predictions were derived from the multilevel logistic model. Vertical dashed lines mark the country-specific medians; facet labels show the country-specific medians and patient numbers.

Predicted ROM-BIV varied between countries, with median values ranging from approximately 0.10 to 0.71 and higher-risk countries showing distributions shifted toward higher malignancy probabilities (Figure 3).

### 3.5. Exploratory Threshold-Based Analyses

To illustrate the mathematical trade-off between malignancy detection and surgical selection, we evaluated model-based performance across a range of threshold probabilities. Across the evaluated threshold range of 15–50%, the proportion of patients classified as above threshold (Supplementary Table S2) progressively declined, while the malignancy rate within this group increased. This was accompanied by fewer false-positive classifications and higher specificity, but also by a progressive loss of sensitivity and a greater proportion of malignancies falling below threshold. These findings illustrate the inherent trade-off between restricting classification to patients with higher predicted risk and maintaining malignancy detection. Detailed estimates are provided in Supplementary Table S2.

**Figure 4:**
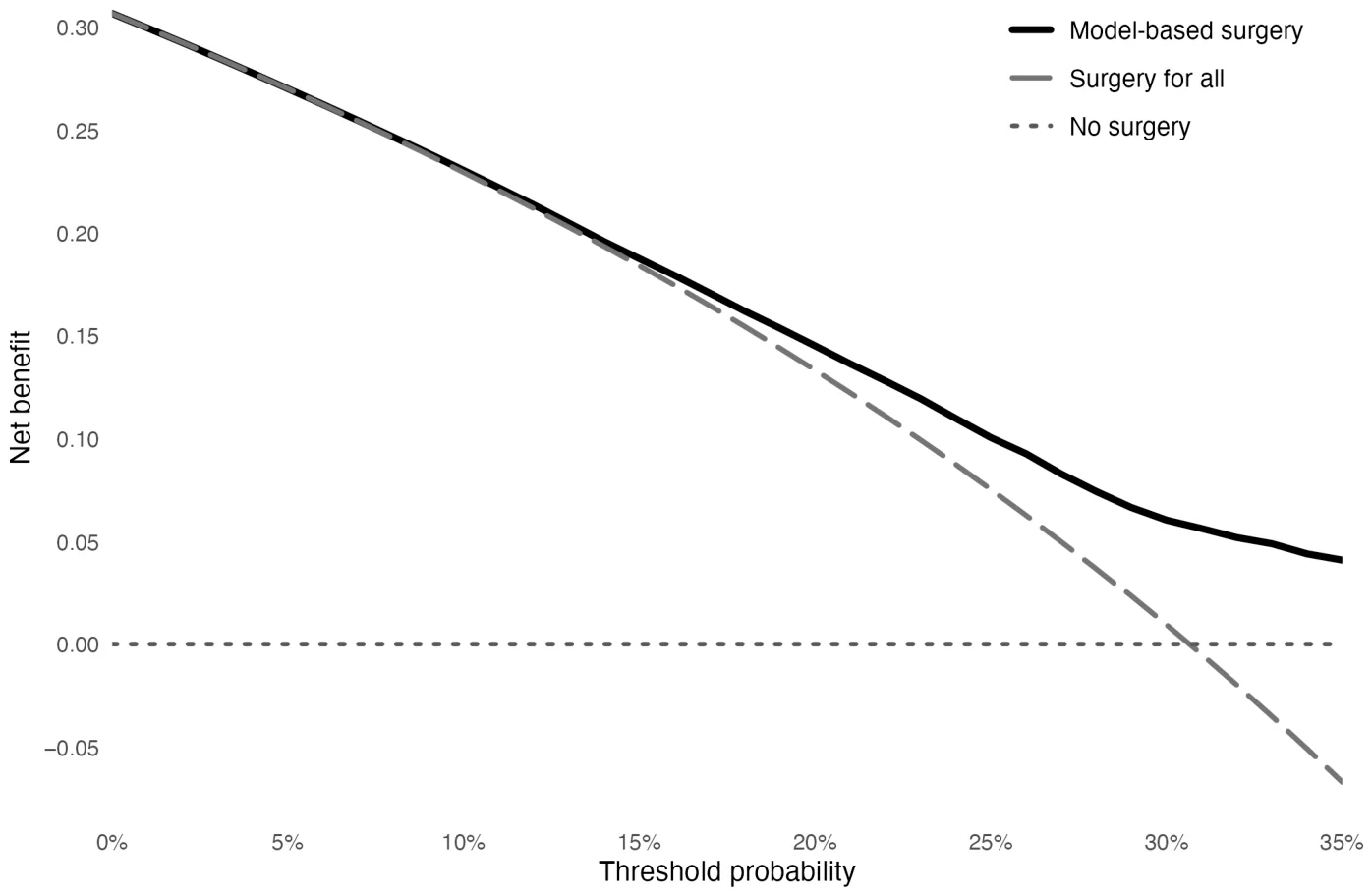
Exploratory decision curve analysis for model-based ROM-BIV prediction of malignancy in the surgically treated cohort. Net benefit is shown across threshold probabilities from 0% to 35% and compared with default strategies of treating all or treating none.

Exploratory decision curve analysis was employed to map theoretical net benefit trends across the evaluated threshold probabilities (Figure 4). Net benefit varied with increasing threshold probability, reflecting the changing balance between false-positive and false-negative classifications. These analyses were intended to illustrate the behavior of the model under different hypothetical thresholds and should not be interpreted as evidence supporting any specific clinical decision threshold.

## 4. Discussion

### 4.1. Institutional Heterogeneity

The overall ROM-BIV within this surgically selected cohort was 30%, consistent with previous registry-based reports [10]. However, this study documents marked institutional variability in ROM-BIV, with model-adjusted center-level estimates ranging from 5.5 to 65.5%. While the 15–50% range frequently reported in the literature is often cited as a benchmark [4,8,9,10], our findings reveal a much more profound variability. This discrepancy suggests that prior estimates, often derived from smaller or more homogeneous series, may have underestimated the true extent of institutional and geographic variance across Europe.

This wide range, which persisted after adjustment accounting for available covariates, suggests that malignancy risk in Bethesda IV nodules is not a fixed biological constant, but a context-dependent parameter. The calculated ICCs (11.4% for institutions and 9.7% for countries) indicate meaningful clustering of ROM-BIV at the institutional and country levels. Sensitivity analyses confirmed that this inconsistency was not merely an artifact of outlying centers; substantial institutional heterogeneity persisted after excluding small centers, extreme centers, and the disproportionately represented Country 10. This indicates that the observed variance is a robust characteristic of the current diagnostic landscape rather than being driven solely by outlying centers or sparse data. Such findings reinforce the classification of Bethesda IV as a diagnostic gray zone and support greater standardization of diagnostic and reporting pathways to mitigate the current context dependency of ROM-BIV estimates.

### 4.2. Potential Drivers of Risk Divergence and Reporting Variability

One potential explanation for the observed heterogeneity is diagnostic variability in ultrasound assessment and cytological interpretation, although this should be interpreted as a hypothesis rather than an established cause. These diagnostic processes, while guided by standardized criteria, remain subject to interobserver differences [17,18,19,20]. Histopathological interpretation, particularly regarding the classification of borderline entities such as noninvasive follicular thyroid neoplasm with papillary-like nuclear features (NIFTP) [21], may further contribute to diagnostic variability. In our cohort, the proportion of papillary thyroid carcinoma (PTC) was notably high (22%). A substantial proportion (39%) of these were reported as follicular variant PTCs (FV-PTCs), which are cytologically consistent with Bethesda IV nodules. However, the remaining PTC cases raise the possibility that incidental microPTCs detected elsewhere in the thyroid specimen were reported as the primary malignancy, potentially inflating ROM-BIV estimates in some centers and making them partly dependent on local reporting standards rather than solely on the malignant potential of the index nodule. Consequently, the significant variance observed across the registry—exemplified by the wide distribution of country-level medians ranging from 10% to 71%—may be partly explained by unmeasured institutional practices and selection biases (Supplementary Table S1).

The impact of such institutional divergence is that comparable patients may have been selected for surgery under different local thresholds. This variability likely reflects fundamental differences in referral patterns, diagnostic work-up, surgical thresholds, and reporting practices among participating centers. Importantly, this institutional heterogeneity also compromises the evaluation of new diagnostic tools or risk-stratification strategies, as differing pretest probabilities can bias performance metrics and limit generalizability of findings. Resolving these inconsistencies is therefore a prerequisite for ensuring that clinical assessments and research data become truly comparable across institutions.

### 4.3. Clinical and Sonographic Factors and the Influence of Surgical Triage

Beyond institutional differences, we examined individual-level predictors within this surgically selected cohort. We observed a statistically significant U-shaped association between age and ROM-BIV (Figure 1), consistent with certain previous reports [22,23]. However, this association must be regarded as descriptive and hypothesis-generating; it may reflect age-dependent clinical triage and surgical selection thresholds rather than a causal effect of age itself. In this context, the higher ROM-BIV observed in younger and older patients may represent a higher clinical suspicion or a more restrictive threshold for non-operative management in these specific groups.

While some previous reports support the utility of TIRADS systems for stratifying malignancy risk in Bethesda IV nodules [23,24,25], others, particularly single-center studies, did not find a significant association [23,26,27]. Our prior EUROCRINE-based study showed that higher EU-TIRADS categories were associated with increased ROM-BIV, supporting the potential value of structured ultrasound risk stratification in Bethesda IV nodules [28]. In the present study, higher EU-TIRADS categories were significantly associated with increased malignancy risk. However, the substantial missingness of EU-TIRADS data in the registry and the model’s limited-to-moderate discrimination restrict the clinical implementation of these findings. Sonographic stratification in Bethesda IV nodules remains a supportive tool, rather than a sufficient basis for individualized management decisions.

Finally, we observed no significant association between registry-reported surgical volume and ROM-BIV, suggesting that institutional case volume alone does not explain the observed variability. These findings reinforce the conclusion that ROM-BIV is a context-dependent metric, necessitating a more standardized approach to preoperative risk assessment.

### 4.4. Exploratory Analysis of Threshold-Based Trade-Off Mechanisms

Our exploratory threshold-based analyses illustrate how different predicted-risk thresholds change the mathematical balance between surgical selection and malignancy detection in Bethesda IV nodules. These projections, derived from a surgically selected cohort, serve as an illustrative mapping of the trade-off mechanism and are not intended to guide management or define clinical cut-offs in non-operated populations. Decision curve analysis suggested variation in theoretical net benefit across the evaluated threshold range, consistent with the changing balance between false-positive and false-negative classifications.

However, these findings do not support implementation of model-guided surgery. The model was not externally validated and showed only limited-to-moderate discrimination; therefore, these results should be regarded as hypothesis-generating rather than as a validated basis for decision-making. Threshold selection remains inherently context-dependent, necessitating careful consideration of local practices, patient preferences, and the specific consequences of a delayed diagnosis in each setting. Consequently, any future risk-based management strategies require rigorous prospective validation in unselected populations before they can be considered for routine clinical use.

### 4.5. Strengths and Limitations

The primary strength of this study is its large-scale, multicenter design, utilizing real-world data from the EUROCRINE registry to evaluate ROM-BIV across diverse European healthcare settings. The hierarchical modeling approach allowed for a robust assessment of individual-level and center-level variance, documenting a degree of systemic heterogeneity that is often obscured in smaller, single-center series. However, several limitations should be considered.

First, because the study is based on a surgically selected cohort, the reported ROM-BIV estimates are conditional on prior clinical triage and cannot be generalized as population-level risk for all Bethesda IV nodules. The documented center-level ICC may partly reflect differences in local surgical selection thresholds, a factor that could not be quantified as non-operated nodules were not captured in the registry.

Second, the voluntary nature of the registry introduces inherent selection biases. The dataset may overrepresent institutions with a specific interest or expertise in thyroid surgery. Furthermore, the overall crude ROM-BIV was sensitive to the reporting volume and case mix of participating countries and centers; this was illustrated by a decrease from 30.7% to 26.9% after excluding the disproportionately represented Country 10. This sensitivity underscores the context-dependent nature of the 30% estimate, which should be viewed as a weighted average of diverse local practices rather than a universal biological constant.

Third, reporting practices regarding incidental microPTCs likely contributed to the observed variability. The registry data did not allow for a reliable distinction between malignancies arising from the index Bethesda IV nodule and incidental findings detected elsewhere in the specimen. Counting such incidentalomas as malignant outcomes may artificially inflate ROM-BIV estimates and vary significantly between institutions, reflecting reporting standards rather than the true malignant potential of the index nodule.

Finally, the substantial missingness of EU-TIRADS data and the lack of other potentially relevant predictors (including nodule size, thyroid autoimmunity, prior radiation exposure, and molecular testing results) limit the model’s ability to account for patient-level risk, as reflected by the limited discrimination of the fixed-effects component (AUC-ROC 0.647).

## 5. Conclusions

This large-scale multicenter study documents substantial between-center and between-country variability in ROM-BIV across Europe, with adjusted center-level estimates ranging from 5.5% to 65.5%. In a surgically selected cohort, this heterogeneity may reflect differences in institutional case selection, diagnostic interpretation, and pathology reporting standards rather than biological differences alone. These findings indicate that ROM-BIV is a context-dependent estimate shaped by the local diagnostic landscape, which compromises the comparability of clinical assessments and research data across institutions.

Clinical and sonographic factors, such as age and EU-TIRADS category, were associated with ROM-BIV. However, our model-based projections must be regarded as illustrative and hypothesis-generating. They demonstrate the mathematical trade-off between reducing the proportion of patients selected for surgery and maintaining malignancy detection, but they do not support the implementation of fixed clinical decision thresholds derived from retrospective registry data.

Despite the limitations inherent in retrospective registries, further exploration of multivariable, model-based approaches may offer a useful pathway forward. Such frameworks could provide a more consistent means of integrating clinical, imaging, and molecular data, thereby helping to mitigate the impact of the institutional heterogeneity documented in this study.

These results underscore the need for standardized diagnostic and reporting pathways to reduce between-center inconsistency. Any future risk-stratification strategy should be positioned as a supportive tool for individualized management and must undergo rigorous prospective validation in unselected populations before clinical implementation can be considered.

## Figures and Tables

**Table 1:** Baseline characteristics of patients with Bethesda IV thyroid nodules.

Characteristic	N = 15,251
Age (years) ^1^	51.0 ± 15.7
Females	12,186 (80%)
EU-TIRADS class	
1–3	2407 (16%)
4	9658 (63%)
5	3186 (21%)
Type of operation	
Lobectomy	10,578 (69%)
Thyroidectomy	3304 (22%)
Other	1369 (9.0%)
Histological main diagnosis	
Follicular adenoma	6703 (44%)
Papillary cancer—other subtypes	2067 (14%)
Nodular goitre	1966 (13%)
Papillary cancer—follicular subtype	1313 (8.6%)
Hürthle cell (oxyphilic) adenoma	1053 (6.9%)
Follicular cancer	793 (5.2%)
Other	757 (5.0%)
Hürthle cell (oxyphilic) carcinoma	293 (1.9%)
Lymphocytic thyroiditis, Hashimoto	188 (1.2%)
NIFTP	118 (0.8%)
Malignant histological main diagnosis	4675 (31%)
Malignant tumor size (mm) ^2^	14 (7–27)
Hashimoto histological main or secondary	2033 (13%)

^1^ mean ± std. dev., ^2^ median (IQR). EU-TIRADS categories were obtained from the first imputed dataset.

**Table 2:** Adjusted risk estimates for malignancy in Bethesda IV thyroid nodules from the hierarchical logistic regression model. Results are based on pooled estimates from multiply imputed datasets. High-volume center: ≥75 thyroid operations per year on average during its participation.

Term	Log(OR)	SE	OR (95% CI)	*p*-Value
Intercept	−1.47	0.30	0.23 (0.13–0.42)	<0.001
Female (vs. Male)	−0.08	0.05	0.93 (0.85–1.01)	0.09327
Age (spline 1)	−0.49	0.10	0.61 (0.5–0.75)	<0.001
Age (spline 2)	1.56	0.44	4.77 (2–11.4)	<0.001
Age (spline 3)	1.22	0.31	3.4 (1.85–6.24)	<0.001
EU-TIRADS 4 (vs. 1–3)	0.28	0.07	1.33 (1.16–1.52)	<0.001
EU-TIRADS 5 (vs. 1–3)	0.47	0.08	1.6 (1.35–1.88)	<0.001
Low-volume site (vs. high-volume)	0.19	0.17	1.21 (0.86–1.7)	0.26593

## Data Availability

Anonymized data will be made available upon reasonable request from the corresponding author.

## Supplementary Materials

The following supporting information can be downloaded at: https://www.mdpi.com/article/10.3390/cancers18193186/s1, Figure S1: Calibration of predicted ROM-BIV. Calibration plot comparing predicted and observed malignancy risk in Bethesda IV nodules, grouped by deciles of predicted probability. The red dashed line indicates perfect calibration. Numerical calibration metrics are reported in the Results section, Table S1: Summary of model-predicted risk of malignancy (ROM-BIV) in Bethesda IV nodules by country, Table S2: Exploratory model-based outcomes across hypothetical ROM-BIV thresholds in surgically treated Bethesda IV thyroid nodules, Table S3: Missing data and imputation procedure for variables included in the main multivariable model, Table S4: Baseline characteristics according to institutional ROM-BIV strata, Table S5: Sensitivity analyses of adjusted center-level ROM-BIV variability.
