# Supplementary material for: Variability in the Risk of Malignancy in Bethesda IV Thyroid Nodules: Results from the EUROCRINE Surgical Registry
Source: Cancers (Basel). 2026 Oct 2;18(19):3186. doi: 10.3390/cancers18193186 (PMC13664412; doi:10.3390/cancers18193186)
Supplement: Supplementary file 1 [file cancers-18-03186-s001.zip › cancers-4555991-supplementary.pdf]

Supplementary Materials: Variability in the Risk of Malignancy in Bethesda IV Thyroid Nodules: Results from the EUROCRINE Surgical Registry

Piotr Wiśniewski , Maciej Śledziński , Marcin Barczyński and Andrzej Rafał Hellmann

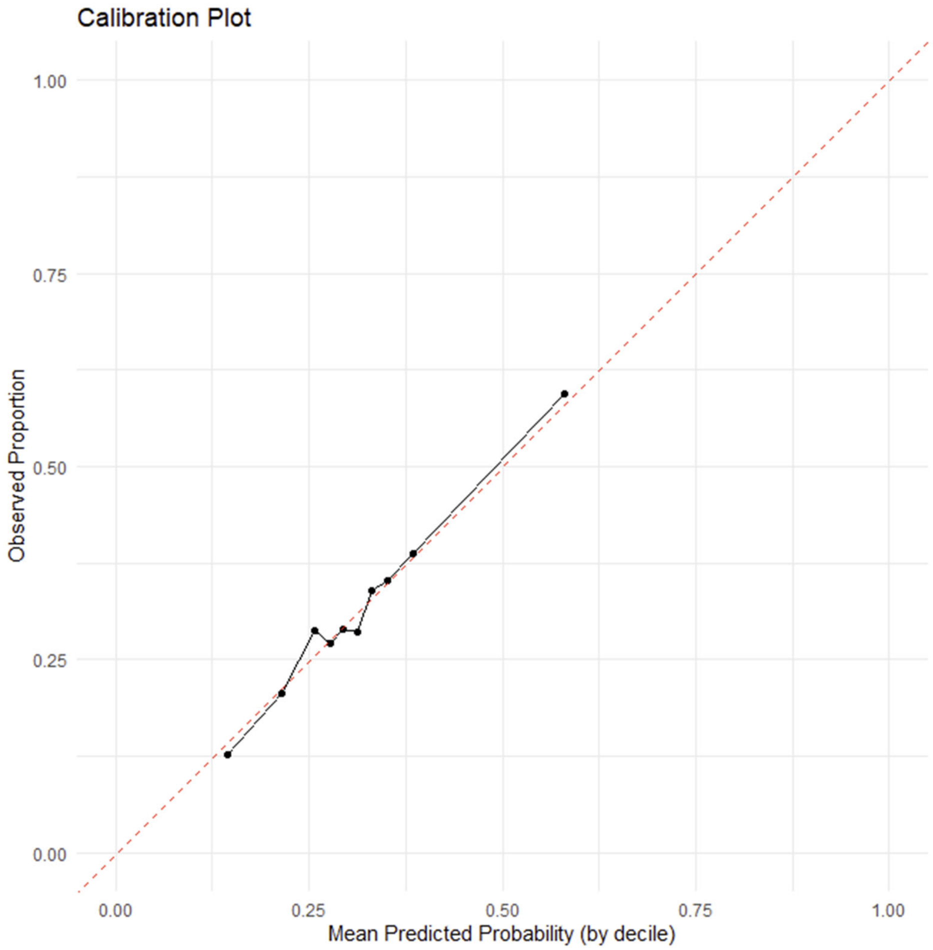

Figure S1. Calibration of predicted ROM-BIV. Calibration plot comparing predicted and observed malignancy risk in Bethesda IV nodules, grouped by deciles of predicted probability. The red dashed line indicates perfect calibration. Numerical calibration metrics are reported in the Results section.

Table S1. Summary of model-predicted risk of malignancy (ROM-BIV) in Bethesda IV nodules by country.

Country ID	Institutions	Operations	Mean	Median	SD	IQR	Min	Max
1	38	2,439	0.263	0.248	0.079	0.099	0.097	0.531
2	2	277	0.170	0.109	0.104	0.188	0.069	0.400
4	10	741	0.250	0.292	0.107	0.180	0.051	0.399
5	5	895	0.400	0.412	0.115	0.091	0.146	0.873
6	4	155	0.730	0.737	0.068	0.089	0.528	0.879
8	18	1,359	0.237	0.227	0.080	0.136	0.075	0.460
9	1	36	0.167	0.171	0.031	0.046	0.107	0.226
10	25	8,340	0.340	0.320	0.110	0.064	0.193	0.880
11	6	616	0.289	0.247	0.128	0.195	0.125	0.631
13	5	393	0.263	0.248	0.066	0.091	0.189	0.491

Values are based on mean predicted probabilities from a multilevel logistic regression model with random effects for country and surgical institutions. SD = standard deviation; IQR = interquartile range.

Table S2. Exploratory model-based outcomes across hypothetical ROM-BIV thresholds in surgically treated Bethesda IV thyroid nodules.

Threshold	% Above threshold	% Malignant among those above threshold	% Malignancies below threshold	Sensitivity	Specificity	Net benefit [1]
0.15	94.5	31.9	0.5	98.3	7.2	0.188
0.20	86.1	33.5	1.8	94.0	17.4	0.145
0.25	76.3	34.9	4.1	86.8	28.4	0.101
0.30	50.0	38.5	11.4	62.8	55.6	0.061
0.35	21.3	47.6	20.5	33.0	83.9	0.041
0.40	10.0	58.3	24.8	19.1	94.0	0.031
0.45	6.0	67.2	26.6	13.2	97.2	0.024

[1] Calculated from decision curve analysis. These analyses are exploratory and should not be interpreted as a validated tool for individualized clinical decision-making. Predictions were based on the fixed-effects component of the model, which showed limited-to-moderate discrimination (AUC-ROC 0.647; 95% CI, 0.637–0.656).

Table S3. Missing data and imputation procedure for variables included in the main multivariable model.

Variable	N observed	N missing	Missing (%)	Imputed	Imputation method
Malignancy status	15,251	0	0.0	No	Not applicable
Sex	15,251	0	0.0	No	Not applicable
Age spline term 1	15,251	0	0.0	No	Not applicable
Age spline term 2	15,251	0	0.0	No	Not applicable
Age spline term 3	15,251	0	0.0	No	Not applicable
EU-TIRADS category	7,550	7,701	50.5	Yes	Multinomial logistic regression
Site volume category	15,251	0	0.0	No	Not applicable
Country identifier	15,251	0	0.0	No	Not applicable
Institution identifier	15,251	0	0.0	No	Not applicable

Note: Multiple imputation was performed using chained equations. EU-TIRADS category was imputed using multinomial logistic regression. Five imputed datasets were generated with 10 iterations.

Table S4. Baseline characteristics according to institutional ROM-BIV strata.

Characteristic	Low-ROM centers	High-ROM centers
N patients	2,928	2,740
Age (years)	54.8 ± 15.6	51.7 ± 15
Females	2,224 (76%)	2,084 (76.1%)
**EU-TIRADS class**		
1–3	648 (22.1%)	676 (24.7%)
4	1,723 (58.8%)	1,601 (58.4%)
5	557 (19%)	463 (16.9%)
**Type of operation**		
Lobectomy	1,939 (66.2%)	1,302 (47.5%)
Thyroidectomy	624 (21.3%)	1,231 (44.9%)
Other	365 (12.5%)	207 (7.6%)
**Histological main diagnosis**		
Follicular adenoma	1,119 (38.2%)	567 (20.7%)
Papillary cancer - other subtypes	205 (7%)	705 (25.7%)
Nodular goitre	772 (26.4%)	436 (15.9%)
Papillary cancer - follicular subtype	43 (1.5%)	187 (6.8%)
Hürthle cell (oxyphilic) adenoma	359 (12.3%)	180 (6.6%)
Follicular cancer	148 (5.1%)	227 (8.3%)
Other	150 (5.1%)	207 (7.6%)
Hürthle cell (oxyphilic) carcinoma	40 (1.4%)	129 (4.7%)
Lymphocytic thyroiditis Hashimoto	65 (2.2%)	43 (1.6%)
NIFTP	27 (0.9%)	59 (2.2%)
Malignant histological main diagnosis	473 (16.2%)	1,309 (47.8%)
Malignant tumor size (mm)	24 (11–39)	14 (8–27)
Hashimoto histological main or secondary	297 (10.1%)	250 (9.1%)

Note: Low-ROM and high-ROM centers were defined as centers in the lowest and highest tertiles of crude institutional ROM-BIV, respectively. Values are mean ± SD, n (%), or median (IQR). EU-TIRADS categories were obtained from the first imputed dataset (.imp = 1).

Table S5. Sensitivity analyses of adjusted center-level ROM-BIV variability.

Scenario	Centers, n	Countries, n	Patients, n	Overall crude ROM-BIV, %	Adjusted center-level ROM-BIV, min %	Adjusted center-level ROM-BIV, median %	Adjusted center-level ROM-BIV, max %	Adjusted center-level ROM-BIV, IQR %
Main analysis: all centers	114	10	15,251	30.7	5.5 (5.3–5.7)	18.2 (17.3–18.6)	65.5 (64.5–65.9)	8.9 (8.3–9.2)
Excluding centers with <20 patients	75	10	14,824	30.7	4.7 (4.6–4.8)	16.9 (16.5–17.1)	69.8 (69.2–70)	9.7 (9.5–9.8)
Excluding centers with <50 patients	40	9	13,667	30.4	4.5 (4.4–4.7)	15.8 (15.2–16.1)	56.2 (55.3–56.9)	9.7 (9.3–9.9)
Excluding centers below 5th or above 95th percentile of crude ROM-BIV	102	10	14,447	29.7	10.5 (10.1–10.7)	17.3 (16.8–17.6)	31.8 (31–32.6)	4.8 (4.6–5)
Excluding centers with <20 patients and centers below 5th or above 95th percentile of crude ROM-BIV	68	10	14,072	29.7	10.3 (10.1–10.6)	16.9 (16.6–17.1)	32.5 (32–33.2)	7.2 (6.8–7.4)
Excluding Country 10	89	9	6,911	26.9	10.6 (10.2–11)	29.7 (28.5–30.7)	74.2 (73.4–74.9)	11.7 (11.2–12.1)

Values for adjusted center-level ROM-BIV are mean estimates across imputations, with the range across imputations shown in parentheses. Country 10 denotes the anonymized country contributing the largest number of patients. ROM-BIV = risk of malignancy in Bethesda IV nodules; IQR = interquartile range.
